# For High Potencies

**Published:** 1897-02

**Authors:** B. Fincke

**Affiliations:** Brooklyn, N. Y.


### FOR HIGH POTENCIES.

(From the Zeitschrift des Berliner Vereins homœop. Aerzte.)

’s muss annersch wer’n.—Wühlhuber, 1848.

In the July number of the *Zeitschrift des Berliner Vereins homœopathischer Aerzte* (Vol. XIV, p. 279), the philosopher of the American Institute of Homœopathy in the beginning of his article: “ Mikrodosisten oder wo ist die Grenze?” (Microdosists, or where is the limit), utters his inability to understand what, in the article “the lone molecule in the 12th centesimal potency” may be meant by the mass from which the molecule is derived which produces the colored lines in the spectroscope. Now molecule is particle of a mass (*moles*, hence the diminutive molecule) of which it forms an integrant part. *Ergo*, as he himself says, “ with the molecule or atom the mass ceases to be,” and consequently also the spectroscopic lines—*i. e.*, the spectroscopic lines represent the mass from which the molecules or atoms are derived. Since the philosopher himself placed the limit of attenuation at the 12th centesimal, which indeed surpasses even the spectroscopic test, the proofs which it furnishes of the presence of medicinal matter can have no value for high potencies.

The insinuation of “continuing the potentiation of the medicinal force after the cessation of the presence of molecular matter,” is not admissible, because such assertion has never been made. The medicinal force of a material dwells in this as the carrier of it aside from its molecularity, which is the firm standpoint which the antagonists of high potencies claim. This firm standpoint, however, begins suspiciously to shake, since the fundamental doctrine of it seriously goes against the fundamental laws of motion, as has been shown elsewhere. The potentiation has no need of this crutch, which forms the firm standpoint for physics and chemistry till something better shall have been discovered than this molecular theory. The calculations of the greatest mathematicians of our time have not at all been decried as psychological trifling, as the philosopher says only the uncertainty has been pointed out, which offers these labors as proofs of the absurdity of potentiation when it steps over the uncertain and wavering limit of the 12th centesimal. "This shall represent exact knowledge," may the philosopher apply to himself. The efficaciousness of the high potencies cannot be cast out by the adaptation of the calculations of even the greatest mathematicians, since it has nothing more to do with the molecularity of matter than that this carries the medicinal force put into it and preserves it. Therefore the whole armamentarium of the materialistic philosophers with their elaborate calculations and astounding observations may be arrayed and by them given as proof of their great erudition, but it cannot change anything in the question to be answered. Mere denial changes nothing, because it is no proof. Even the vilest criminal dies rather than to confess his guilt. The true way is the philosophical one of induction. If this, in science, is the only infallible instrument for discovering the truth in difficult questions, why should it be discarded and lose its value when the science of homœopathics is concerned?

Instead of traveling this indeed difficult and often tedious path, the adversaries of the high potencies, though they claim the name of homœopathists, follow the lead of those who, under the pretense of being scientific, combat potentiation, or try to kill it by silence. But this stratagem begins to lose its force, because " the sparrows on the roofs already talk about it," and at last, after more than fifty years, the high potency of Hahnemann—for he was the first to prepare and give it its name—has penetrated the thick skin of the scientific rhinoceros so that it stands at bay and precipitates itself directly upon its enemy. But there is no enemy. The high potency acts in the clumsy body of the monster against its will. In one word, the high potency exists and has come to stay.

The Hahnemannian way of induction, therefore, is the only one which can be used to answer the question. This is the way of experiment which Hahnemann has recommended in the words: "*macht's nach, aber macht's genau nach*" (do as I did, but exactly so). The experiment demands first, that we procure the pure medicinal substances, and then carefully subject them to comminution, according to the rules which he has given. Now how could Hahnemann have found that this comminution develops greater medicinal force than was present in the original substance, or that on the contrary it makes out of poisons remedies? Perhaps by consulting the microscope or the spectroscope, which in his lifetime did not yet exist! Or by calculations of the greatest mathematicians of his time relating to the infinitesmals which compose the medicinal matter? Or by the occult sciences cultivated by the Middle Ages? Nothing of all that! He simply gave his remedies to the healthy person and observed their actions in them, and wrote them carefully down. This was one part of the induction, by which he learned the pure actions upon the healthy, and by it he created the pure materia medica. Progressing in these experiments, he certainly arrived at the limit appointed by one of his epigones to be the 12th centesimal potency. But knowing nothing of this future limit, he continued his potentiation and arrived at the 30th centesimal, which, for a wonder, is acknowledged by all good disciples of Hahnemann, though they rail against the transference of medicinal force by means of and upon inert vehicles. Here he had reached already a scale of 30 degrees, and was enabled to institute comparisons which moved him to recognize in the 30th centesimal potency sufficient strength to serve as well for proving the remedies upon the healthy as for healing the sick. This, then, was the second part of his induction. If the potencies which he obtained by comminution and elimination of the substance containing the medicinal force were able to distune the life-force, they must also be capable of healing, where applied to the sick according to symptom-simility. The result taught him the correctness of his induction, and the thousands after him blessed his memory, because he has taught them to prepare the remedies in such a manner that they exert their healing power, which is expected from them, without doing any harm. This is the homœopathic proof of the efficaciousness of the homœopathic healing potencies.

What objections can the opponents make to this homœopathic argument? Why do they not go to work themselves—if they want to honor the truth (p. 261)—in order to convince themselves that, as they say, "the most irrevocable, the most overwhelming proofs are extant for the efficaciousness of the infinitesimal doses," as the thousands and thousands of experiments of the high potentialists teach since Hahnemann's time? The proposition that every homœopath should make one to three experiments with high potencies on chronic patients is acceptable, but at the same time it shows the insufficiency of such slow and reluctant progress. Why not make experiments in acute diseases? There are always plenty of such cases on hand, and they promise a more speedy and multiple experience, apt to strengthen the confidence in high potencies. The proposition, alas! looks too much like an effort to render the cause of high potencies a chronic disease of Homœopathy. We, who have not long enough to live till all the homœopathic skeptics have made their yearly one to three experiments on chronic patients for their own satisfaction cannot wait for them. It, indeed, seems that even the arguments in favor of high potencies are only turned to their disadvantage. That along with them, even macrodosia is favored, shows what may be expected of such impartial homœopathists, namely—nothing. After the neuro-analytical experiments of high potencies which denote a new era in homœopathics which proved the efficaciousness of the 4,000th centesimal potency, nothing followed but a fending off: "Wozu?" (to what purpose). One was content with one's success with low potencies. The splendid experiments were grudgingly acknowledged, and later on ignored and silenced in the homœopathic ranks, and afterward the allopaths blamed for it. And yet the neuro-analytician was on their own side, for he declared before the Association of Naturalists and Physicians in Salzburg that with the neuro-analytical proof of the efficaciousness of the 4,000th potency (cent.) the divisibility of matter must be extended that far. He, therefore, evidently was on the side of the materialists, and could, perhaps, not judge differently on his standpoint as naturalist. But as a physician he had to consider that his neuro-analytical reaction did not support the infinite divisibility of matter, but signified the reaction of the life-force of the analytician to the immaterial dynamic potency. This reaction can, according to electro-magnetic neural analysis, be indicated by any high potency high above the 4,000th centesimal. This, however, oversteps the limit of the present science, and is therefore also under the ban of deadly silence, for it undermines likewise the fundamental elements of this science just as much as the proof of the incompatibility of the molecular theory with the fundamental laws of motion.

The capital which the opponents of high potencies still draw from the notion of infinite divisibility of matter, is alas! like the Confederate money in this country, not worth anything. This idea, which still haunts the homœopathic materialists, has long been given up by the high-potentialists. *Sunt certi denique fines.* Theoretically, it is long ago discarded by the molecular theory, for this indicates a limit to the minuteness to which the matter can arrive, though also here reigns the same uncertainty as with us about the limit of the efficaciousness of high potencies. But with *their* uncertainty we have here nothing to do. We already get rid of the matter when we commence potentiation, for we do not potentiate the matter, but the inert vehicle which by the potentiation we render medicinally active. The medicinal force is not subject to the laws of physics and chemics, but to the laws of the life-force which these departments of science, including physiology, have thrown out of their temples. From the Hahnemannian life-force which comprises the whole organism, they have come down to the nerve-cell which now is to explain all phenomena of life. Poor creatures! would Hahnemann exclaim. We, then, do not need the concept of infinite divisibility of matter for the explanation of the high potencies, and rather propose an infinite divisibility of force which it will not be so easy to deny than that of matter. The Hahnemannian use of matter as vehicle, carrier or menstruum of the force assigns the proper place to it. It refutes the senseless assertion that the force could not be separated from matter, and that it was against all reason to speak of transference of force (p. 259). If the advocate for and against high potencies who denies it would hit his head against an open door as it sometimes happens in the dark, he no doubt would become aware of something like transference of force. For his motion against the door is reiterated by the shock which this exerts. The force with which he hits the door is transferred upon it for the moment, and, since the door cannot escape, is given back by it and re-transferred. The doubt of the transferability of force is most astonishing, for every motion in the world is necessarily a transference of force. Motion is nothing in itself, it will always be a transference of force upon matter. Why should it then be different with the medicinal force? The physical and chemical force is not a medicinal force. This medicinal force is something *sui generis*, and has only relation to the life-force, by which alone it is to be recognized, the matter being only the vehicle for it. This, of course, is incomprehensible to those who deviate from Hahnemannian principles in order to hasten after the modern natural science. The ideas laid down in the first thirty paragraphs of *The Organon* are antiquated in their minds, and no more adequate to the refined notions of the *fin de siecle.* As if ever true ideas could lose any of their value and freshness!

And yet the philosopher of the American Institute and the advocate for and against are of one mind that it must become "otherwise" (p. 279), and that the infinitesimal doses must be cast out into utter darkness (p. 276), where there is wailing and gnashing of teeth. So far, then, has it come at the end of this glorious Hahnemannian century that the high potencies must go the way of all flesh, as the physico-chemical school has done with the life-force. One should not think it possible that men who are constantly talking of science and scientificness, could so recklessly violate the rules of science and of decency that they think of doing away with questions the solution of which is so strange and impossible to them, by simply throwing them out and killing them by silence afterward. That is a beautiful *remedium* (§ 277) of an evil which drags itself along ever since the schism of Trinks without leading to anything else than to thrash the same old empty straw over and over again. Do they not even think of it that, if driven to the wall by the high potencies, they declare for macrodosy, they give the greatest aid and comfort to the enemy, the macrodosy of which is not at all to be compared to that in Homœopathy? Do they not remember how many fall victims to that allopathic macrodosy, the inoculation of morbid matters included; and has the advocate for and against to offer against the just demand to abolish this poisonous macrodosy nothing else than to claim that the infinitesimal doses must be thrown out of Homœopathy? (§ 276). The rage against high potencies is so great that they forget altogether that the doses of the homœopathic materialists are still small in proportion to the allopathic doses, may they be ever so incomparable to the high-potential doses? But according to our conceptions the magnitude of the doses has nothing to do with their efficaciousness, since this follows not the matter but the medicinal force carried by it, and depends upon the necessary proportionality of the remedy to the life-force, which again depends upon the symptom-simility of the remedy, and the sensitivity of the life-force. Should chemistry be held responsible for it, however, then it militates against the Hahnemannian doctrine and is a *confusio idearum.* This indeed means to allow to the spirit of party too much play-room, with which the high-potentialists have nothing whatever to do. They alone stand upon the firm ground of the Hahnemannian homœopathics which we recognize and acknowledge in the first part of *The Organon.* To our knowledge Hahnemann has not yet been abolished by the enemies of high-potencies, though they have kept nothing from his doctrine than the symptomsimility, and even this stronghold is curtailed and called in question and discredited if it finds its application in the high-potency question.

Then they doubt their own observations, and, according to the adage: "*Was ich denk und thu, trau ich andern zu*" (what I think and do, I do think of others too) also those of others. Then they betray by their provings with nothing as they expect milk-sugar to be, the provers and themselves and explain the phenomena and symptoms in others not proved by them, by suggestion, credulity, of both prover and observer and general skeptics which they think to escape by throwing themselves in the arms of physics and chemistry and even mathematics, in departments of science of which they themselves confess not to be experts, from which though they expect the salvation for homœopathic posology. These "*Wühlhubers*" (revolutionaries) in Homœopathy in their rage, hurry to the hostile camp, and the "*anders werden*" (becoming otherwise) relates only to the emasculation of the homœopathic body in order to make it acceptable to general medicine—*i. e.*, to the allopathy or physico-chemical school of medicine. What can the molecular theory have to do with the homœopathic healing art and science? Nothing. They are two quite different fields which the two schools, the homœopathic and the physico-chemical, are cultivating. The one should not be mixed with the other. The homœopaths should not forsake the Hahnemannian doctrine which makes potentiation complementary to the symptom-simility. If one reads the tirades of these impartial advocates for and against, and what these outspoken opponents of potentiation put in the mouths of high potentialists, one should think they would understand that matter in all its bearings. But not at all. They confess themselves, without blushing, that practically they know nothing about it, as the American teachers at the American colleges did, who—22 of 28—declared publicly in the session of the American Institute last year that they teach nothing about high potencies because they know nothing about them, and do not know the laws according to which they are to be administered. Among these were not even the great lights of this institution, who wisely let their light shine in the background. And in Germany it is not different also, with very few most honorable exceptions. After all the learned disquisitions they arrive at the same conclusion, and even go further, to get the whole high potency question, *par ordre du mufti*, out of the world. Oh! the Pharisees and Scribes who strain at the gnats and swallow camels!

"Microdosists, or where is the limit?" So asks the philosopher of the American Institute. How wonderful! After having placed the boundary-post at the 12th centesimal, he asks the microdosists—for of the others he can naturally not expect an answer—where is the limit? The limit of what? Of the matter? That limit he himself already has settled with the aid of the greatest mathematicians of our times. Has he, as the advocate for and against (p. 275) apprehended, sawn off the branch upon which he was sitting, and suffered therefrom an unkind fall? The homœopathic argument, which he had neglected by shifting the question upon a strange field, that of the physico-chemical school, has made him forget that the branch is connected with the tree. When this tree grows, how can he predict where the limit of its growth will be? The trees certainly do not grow into the heaven, they have quite a distinct limit. Such is also the case with the matter, the limit of which has not yet been found in spite of all calculations and theories. And so it is also with the limit of the medicinal force, the limit of which we still seek, for in the 13,000,000th potency of Lachesis the action of this remedy is still distinctly recognizable. Some opine that there is no such limit, but I cannot admit this as long as the experiment shows action. My opinion is that there is such a limit, and that we must potentiate till no more action can be discerned. The philosopher, following the experience of the physico-chemical school, has stopped at the 12th centesimal. Of that, what lies beyond this limit, he is not entitled to judge if he has not made the experiment. His doubt and refutation, then, has no value, and cannot stop the continued development of homœopathics in this direction. The adversaries forget or neglect that the simility is just as well necessary for the dose as for the symptoms. The dose must be similar to the reaction of the organism or its life-force. Everywhere where Hahnemann speaks of symptom-simility he makes the least possible dose the condition of a successful result. The high potencies confirm the Hahnemannian doctrine. This cannot "*anders werden*" (change).

We will now look a little nearer at the wisdom of the advocate for and against high potencies (p. 276):

"The materiality of the high potencies is still unproved." Naturally it will remain so in all eternity, because a potency is only a force which is carried by the inert material vehicle, but not matter itself.

"Force without matter exists nowhere in nature." Of course not, because the force needs matter as a supporting vehicle, the mechanical force as well as the physical and chemical, and besides also the medicinal force.

"The medicinal force is dependent exclusively upon the chemical constitution of the medicinal body, it is ingrown in it." This assertion is denied by the potentiation and disproved by the action of the high potencies on the healthy and sick, according to the homœopathic argument. Behold here the physico-chemical foundation of this so-called Homœopathy! Hahnemann has already in his lifetime condemned it relentlessly as the origin of the pernicious mingling sect. The high potencies have justified his teaching gloriously, and hiding under the wings of the science which at the present time is carried on by the materialists, cannot make it otherwise.

"This medicinal force cannot be separated from the medicinal body nor transferred upon other bodies." This is a mere opinion contradicted by every fact before our eyes. For forces are constantly transferred from one body to another, as well in mechanics as in physics and chemistry. That forces can also be separated from medicinal substances and transferred upon other inert substances in Homœopathy Hahnemann has already taught us, and already the few potencies up to the 12th potency, which these gentlemen on the other side acknowledge, prove the correctness of his observation. For nowhere have they shown that it was the chemical force of the last Mohican of a molecule in this potency which effected a proving or cure. The chemical force, is, of course, only that force which by the affinity of the matters effects their separation or combination, processes which again proceed by a transference of forces. However, the *modus operandi* of low potencies, even of the 30th centesimal, which they acknowledge has nowhere and never been tried by these homœopathic materialists, except by that bastard of Homœopathy—the Schüssler method, and by the efforts of Grauvogl. How do they explain the chemical action of even that notorious 12th centesimal potency in which possibly still a (calculated) molecule of the original substance may linger? This, these chemical philosophers must explain and demonstrate clearly, before they dare to throw the high potencies overboard.

"The observation of successful healing after homœopathic therapeutic doses cannot admit a safe conclusion backward upon the previously-used remedy as cause." This sentence is utterly false. If according to this assertion other experiments in physics and chemistry are to be judged of, they would be of just as little value as the therapeutic successes are said to be. The examination of a body by the chemist can certainly teach only by the result (the chemical success) what it is. The advocate says (p. 269): "an empirical fact is no proof." How can that be? The success is a fact and a fact furnishes always a proof. This the advocate proves himself. Since he has not to show any successful healing after therapeutic doses of high potencies, he doubts that others like him could not have had such successes, and this doubt again is just as little a proof, since it is founded upon no fact. The abuse with the "*post hoc ergo, propter hoc*" comes again here likewise to the fore, and yet we don't know anything *propter hoc*, when we have not experienced it *post hoc*. Or will the advocate not acknowledge the supreme value of the experiment, because it tries to find out what previously was unknown? There is no surer proof than the homœopathic argument based upon empirical facts, in order to admit the conclusion backward from the action of a remedy, high or low potency, upon the organism, a proof which of course must have also scientific validity, for the science is, it is to be hoped, not an exception to the common logic? Of many remedies, we know through the provings of Hahnemann on the healthy, that they have ever-recurring, distinctly-recognizable symptoms. If these symptoms occur in the sick, they will disappear after the administration of the similar remedy. This is the only art-cure that may exist which at the same time satisfies the scientific requirement if, provided that the experiment was pure.

With artificial, self-made difficulties as the advocate creates in order to discredit such results, nothing can be gained. A good homœopathic physician knows well to discriminate between art-cure and cure by nature. But with the expression art-cure also frequently an improper meaning is connected, inasmuch as it is understood by this term that the cure has been effected homœopathically, according to the allopathic rules. After all, all art-cures are but cures of nature, and since THESE fulfill the desired purpose, to restore health, they are worth just as much as the art-cures, which have been effected at the expenditure of an enormous pathological erudition. Works of art are not created according to the prescribed rules and laws of science, but they originate out of the spirit of the artist, poet, sculptor, painter. It is just the same with the healing artist. If the materia medica were perfect and easy of access, every one would be enabled to see at a glance how art and science united in the production of the success gained empirically or by art. The greatest scholars have not always at the same time been the greatest artists.

This conclusion backward from the high-potency to the constitution of matter is of the higest importance. For through it the Hahnemannian potentiation, of which the high-potencies are only the necessary consequence, has become a philosophical instrument, which finds its application and use in all departments of knowledge. Hahnemann was far ahead of his time, and, though misunderstood, even by many of his adherents, still reaches far into the coming centuries. How long shall it last, till the medical profession accepts the conception of things as Hahnemann represents, and makes it the motive of its medical action, if active and intelligent members in our own ranks do not shrink from the impudence to extend their clinched fist to the progress, and to force it to be content with the satisfactory results which this Hahnemannian conception vouchsafes in thought and action. May these adversaries try ever so much to calumniate the cause of high potencies, to abuse and ridicule it, they will never succeed to kill it. It is too late for that. Majorities and despotic power can indeed retard the progress of the spirit for a time, but the truth breaks through the restraint again victoriously, because it passes from spirit to spirit, according to the eternal law of transference of force.

*Ceterum censeo macradosiam esse delendam.*

B. Fincke, M. D.

Brooklyn, N. Y., September 29th, 1895.

